# Combining Metabolic Analysis With Biological Endpoints Provides a View Into the Drought Resistance Mechanism of *Carex breviculmis*

**DOI:** 10.3389/fpls.2022.945441

**Published:** 2022-07-07

**Authors:** Zhaorong Mi, Yingying Ma, Pinlin Liu, Haoyi Zhang, Lu Zhang, Wenqing Jia, Xiaopei Zhu, Yanli Wang, Chan Zhang, Lin Du, Xilin Li, Haitao Chen, Tao Han, Huichao Liu

**Affiliations:** ^1^School of Horticulture and Landscape Architecture, Henan Institute of Science and Technology, Xinxiang, China; ^2^Henan Province Engineering Research Center of Horticultural Plant Resource Utilization and Germplasm Enhancement, Xinxiang, China; ^3^College of Life Sciences, Henan Normal University, Xinxiang, China

**Keywords:** *Carex breviculmis*, drought stress, amino acid, biological endpoint, metabolic profiling, TCA cycle

## Abstract

Metabolomics is an effective tool to test the response of plants to environmental stress; however, the relationships between metabolites and biological endpoints remained obscure in response to drought stress. *Carex breviculmis* is widely used in forage production, turf management, and landscape application and it is particularly resistant to drought stress. We investigated the metabolomic responses of *C. breviculmis* to drought stress by imposing a 22-day natural soil water loss. The results showed that water-deficit restrained plant growth, reducing plant height, leaf fresh weight, and total weight, however, increasing soluble protein content and malondialdehyde content. In total, 129 differential metabolites in the leaves were detected between drought and control using the Ultrahigh Performance Liquid Chromatography-Mass Spectrometer (UPLC-MS) method. Drought enhanced most of the primary and secondary metabolites in the differential metabolites. Almost all the sugars, amino acids, organic acids, phytohormones, nucleotides, phenylpropanoids and polyketides in the differential metabolites were negatively correlated with plant height and leaf fresh weight, while they were positively correlated with soluble protein content and malondialdehyde content. Metabolic pathway analysis showed that drought stress significantly affected aminoacyl-tRNA biosynthesis, TCA cycling, starch and sucrose metabolism. Our study is the first statement on metabolomic responses to drought stress in the drought-enduring plant *C. breviculmis*. According to the result, the coordination between diverse metabolic pathways in *C. breviculmis* enables the plant to adapt to a drought environment. This study will provide a systematic framework for explaining the metabolic plasticity and drought tolerance mechanisms of *C. breviculmis* under drought stress.

## Introduction

Water accounts for a significant part of the fresh weight of herbaceous plants and plays a vital role in plant growth, development and metabolism (Lisar et al., 2012). Drought is one of the most common and critical environmental stresses for plants in many world areas, which is multi-dimensional stress and leads to variations in the physiological, morphological, biochemical, and molecular traits of plants (Bhargava and Sawant, 2013). Drought stress could induce the skyrocket of reactive oxygen species, including free radicals (superoxide, alkoxy, and hydroxyl radicals) and non-radicals (singlet oxygen and hydrogen peroxide). Those are fairly toxic and disrupt cellular homeostasis by destroying proteins, carbohydrates, lipids, and DNA (Anjum et al., 2017).

Drought not only externally affects morphological characteristics (e.g., plant height, canopy, root development and leaf area index), but also internally affects plant physiology (e.g., osmotic potential, transpiration rates, carboxylation efficiency and photosynthesis rate; Farooq et al., 2012; Davis et al., 2014). These effects are achieved by altering plant metabolisms. However, few studies reported the influence of drought stress on metabolic pathways. According to Michaletti et al. (2018), drought has a significant effect on metabolic pathways, and the number and variety of impacted pathways vary among different plants. Therefore, the drought-induced metabolic pathways remained large uncertainties and need further investigation. The changes in metabolic pathways under drought significantly affect the content and varieties of metabolites. For example, some amino acids demonstrated prominent increase under drought stress, such as allantoin, proline, arginine, histidine, isoleucine and tryptophan. However, the changes of some metabolites are not consistent under drought stress among plants and studies. For instance aspartic acid was elevated under drought in some studies (Haghighi et al., 2020) while decreased in others (Khan et al., 2019). Such a discrepancy under drought stress was also found in 2-oxoglutarate (Zhang et al., 2021a vs. Silvente et al., 2012), malic acid (Lahuta et al., 2022 vs. Ashrafi et al., 2018), gulose (Erxleben et al., 2012 vs. Rizhsky et al., 2004), succinate (Zhang et al., 2021b vs. Hamanishi et al., 2015). The changes in these metabolites and pathways ultimately lead to changes in external and internal biological endpoints of plants. However, the relationship between changes in metabolites and biological endpoints under drought remained unclear.

Genus *Carex*, consisting of over 2000 species of the family *Cyperaceae*, are widely distributed in temperate and cold regions (Ning et al., 2014). According to the research of Yang et al. (2014), *Carex breviculmis* is particularly resistant to drought stress among the 21 *Carex* species in Northern China, providing suitable research material for drought resistance mechanism. Hou et al. (2021) found the antioxidant enzymatic activity and osmotic adjustment are components of the drought tolerance mechanism in *Carex duriuscula*. However, the changes of morphology, physiology and metabolic pathways and the relationships between the variation of metabolites and biological endpoints under drought stress in *C. breviculmis* are poorly understood.

In this study, *C. breviculmis* was treated under drought stress. The aims of our study were (1) to assess the potential impact of drought stress on the growth and development of *C. breviculmis*, (2) to offer a global view of the drought tolerance mechanisms of drought stress by combining the metabolic analysis and biologic endpoints [e.g., plant height, leaf fresh weight, soluble protein content and malondialdehyde (MDA) content], and (3) to illustrate the metabolic pathway bridging the changes in metabolites and biologic endpoints under drought stress.

## Materials and Methods

The *C. breviculmis* was obtained from Institute of Grassland, Flowers and Ecology, Beijing Academy of Agriculture and Forestry Sciences. Plants of the same size were transplanted into plastic pots with an upper diameter of 189 mm, bottom diameter of 160 mm and height of 195 mm in March, 2021. The substrate was obtained by sieving the upper 20 cm depth of farmland (alluvial soil) through a 2-mm sieve. Each pot filled with 4.5 kg of soil. Plastic pots were placed in a greenhouse on the campus of Henan Institute of Science and Technology. Each treatment contained ten pots, with 40 plants per pot. On September 20th, the drought treatment ceased irrigation for 22 days to simulate the drought stress, while the control watered every 2 days.

### Growth Attribute Assay

Four pots were randomly selected to measure plant height, crown breadth, leaf length and leaf width every 7 days after applying drought stress. On the 22nd day after the treatment application, the fresh and dry weight of leaves and roots were measured for four pots. For dry weights, leaves and roots were oven-dried at 65°C to constant weight.

### MDA, Superoxide Dismutase Activity, Soluble Protein Content and Proline Content Assay

Leaf sample (0.5 g) was randomly selected in each pot and ground in 10 ml cold phosphate buffer (62.5 mM, pH 7.8), followed by 10 min centrifugation at 15,000*g*, 4°C to get supernatant. The supernatant was used to determine the SOD activity and MDA content following the method of Han et al. (2019). Leaf tissue (0.2 g) was ground in 5 ml distilled water and centrifuged at 10,000 r/min, 4°C for 10 min to obtain supernatant. The soluble protein content was determined according to the process of Read and Northcote (1981) using the reagent Coomassie Brilliant Blue G250, followed by absorbance measurements at 595 nm using bovine serum albumin as standard. Proline was extracted from leaf tissues (0.2 g) and determined spectrophotometrically at 520 nm (Bates et al., 1973). Each parameter was performed in quadruplicate every 7 days.

### Metabolite Analysis

On the 22nd day, the randomly selected leave tissue (0.2 g) was deactivated with liquid nitrogen and ground in a 2.0 ml extract solution (methanol:water = 3:1). Then, the samples were homogenized and sonicated in an ice-water bath for 5 min with 3-times repetition. Then the samples were incubated for 1 h at −40°C and centrifuged at 13,800*g* (4°C, 15 min) for supernatant. LC–MS analyses were carried out using an ultra-high-performance liquid chromatography system (Vanquish, Thermo Fisher Scientific) coupled to Q Exactive HFX mass spectrometer (Orbitrap MS, Thermo). Metabolites were annotated by automatically comparing to retention time, ion feature and tandem mass-spectrometry fragmentation pattern of an in-house database. The metabolite analysis was performed in quadruplicate.

### Statistical Analysis

R v. 4.1.3 (R Core Team, 2022) was used for statistical analyses. SIMCA 14.1 (Sartorius, Gottingen, Germany) was employed for orthogonal partial least squares regression analysis. The heatmap was done by MetaboAnalyst through hierarchical clustering and average linkage clustering.

## Results

### Morphological Changes Under Drought Stress

**Figure 1:**
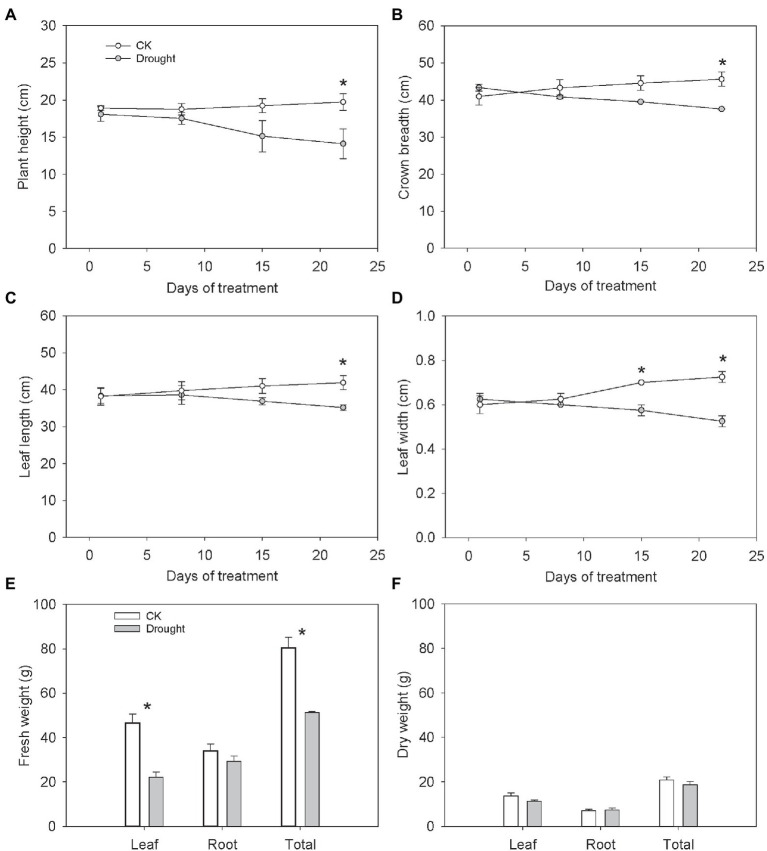
Growth characters of *Carex breviculmis*. **(A)** Plant height. **(B)** Crown breadth. **(C)** Leaf length. **(D)** Leaf width. **(E)** Fresh weight. **(F)** Dry weight. Asterisk (*) denotes a significant difference at *p* < 0.05. Vertical bars indicate standard error (*n* = 4).

The plant height, crown breadth, leaf length and leaf width decreased with the drought aggravation (Figures 1A–D). After 22 days of treatment, the plant height, crown breadth, leaf length and leaf width significantly declined under drought stress by 28.52, 17.62, 16.05, and 27.59%, respectively (Figures 1A–D). Drought stress significantly reduced the leaf fresh weight and total fresh weight by 52.47 and 36.24%, respectively (Figure 1E), indicating adverse effects of water deficit on plant growth. However, the leaf dry weight, root fresh weight, root dry weight and total dry weight showed a non-significant change (Figure 1F).

### Influences of Drought Stress on MDA Content, Soluble Protein Content, Proline Content, and SOD Activity

**Figure 2:**
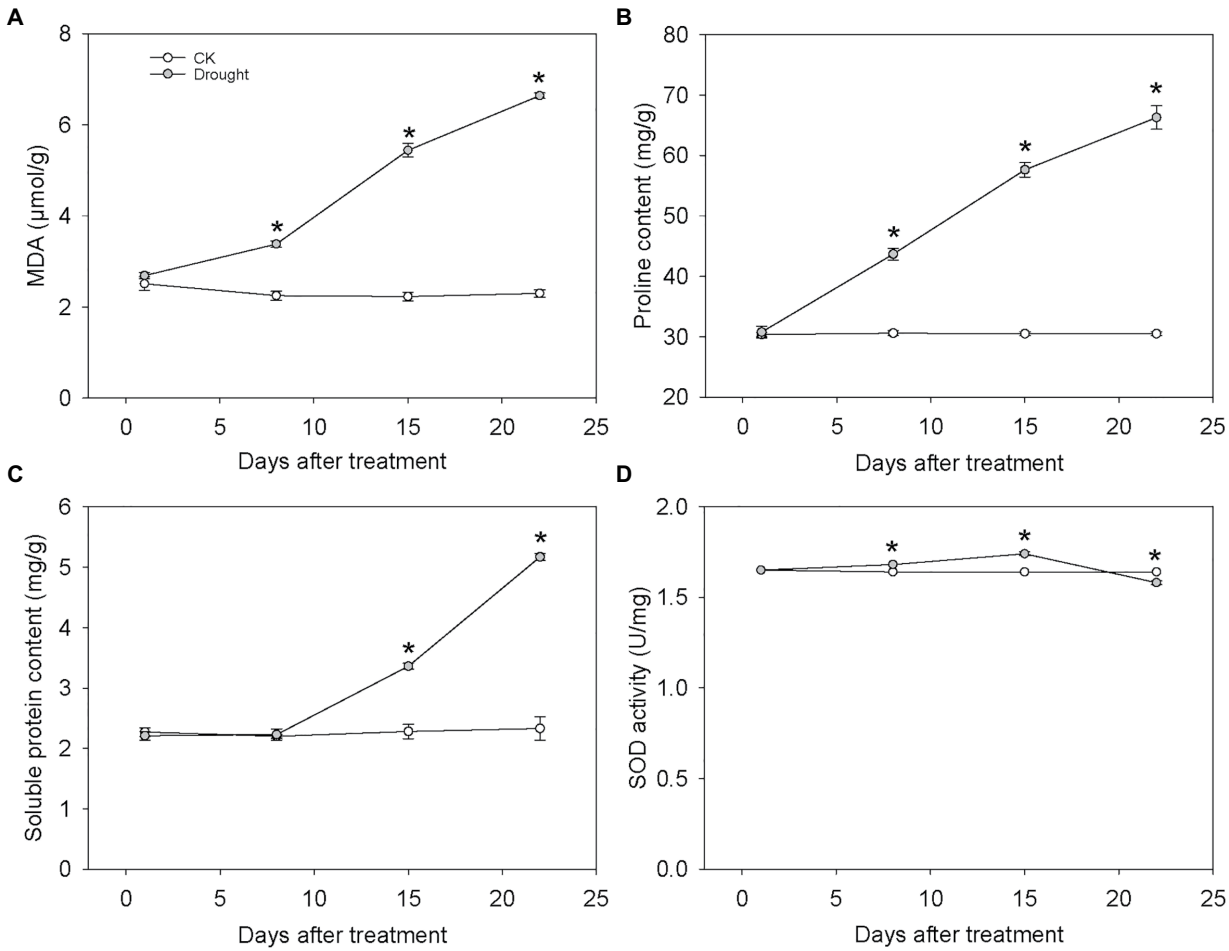
The MDA content, Proline content, Soluble protein content and SOD activity of *C. breviculmis*. **(A)** MDA. **(B)** Proline. **(C)** Soluble protein. **(D)** SOD activity. Asterisk (*) denotes a significant difference at *p* < 0.05. Vertical bars indicate standard error (*n* = 4).

The content of MDA, soluble protein, proline in leaf under drought stress increased as the drought aggravated. However, the SOD activity rose firstly and then fell at the end of the experiment. The MDA content of leaf significantly increased by 50.29, 143.71, and 189.09% at 8, 15, and 22 days under drought treatment, respectively (Figure 2A), indicating the drought-induced lipid peroxidation exacerbated as the water deficit aggravated. Similarly, the proline content significantly increased under drought treatment by 42.86, 88.90, and 117.24% at 8, 15, and 22 days, respectively (Figure 2B). Compared with control, the soluble protein content under drought stress significantly increased by 47.40 and 122.01% at 15 and 22 days, respectively (Figure 2C). The SOD activity under drought stress significantly increased by 2.03 and 5.69% at 8 and 15 days, but significantly decreased by 3.46% at 22 days under drought treatment (Figure 2D).

### Metabolite Changes Under Drought Stress

**Figure 3:**
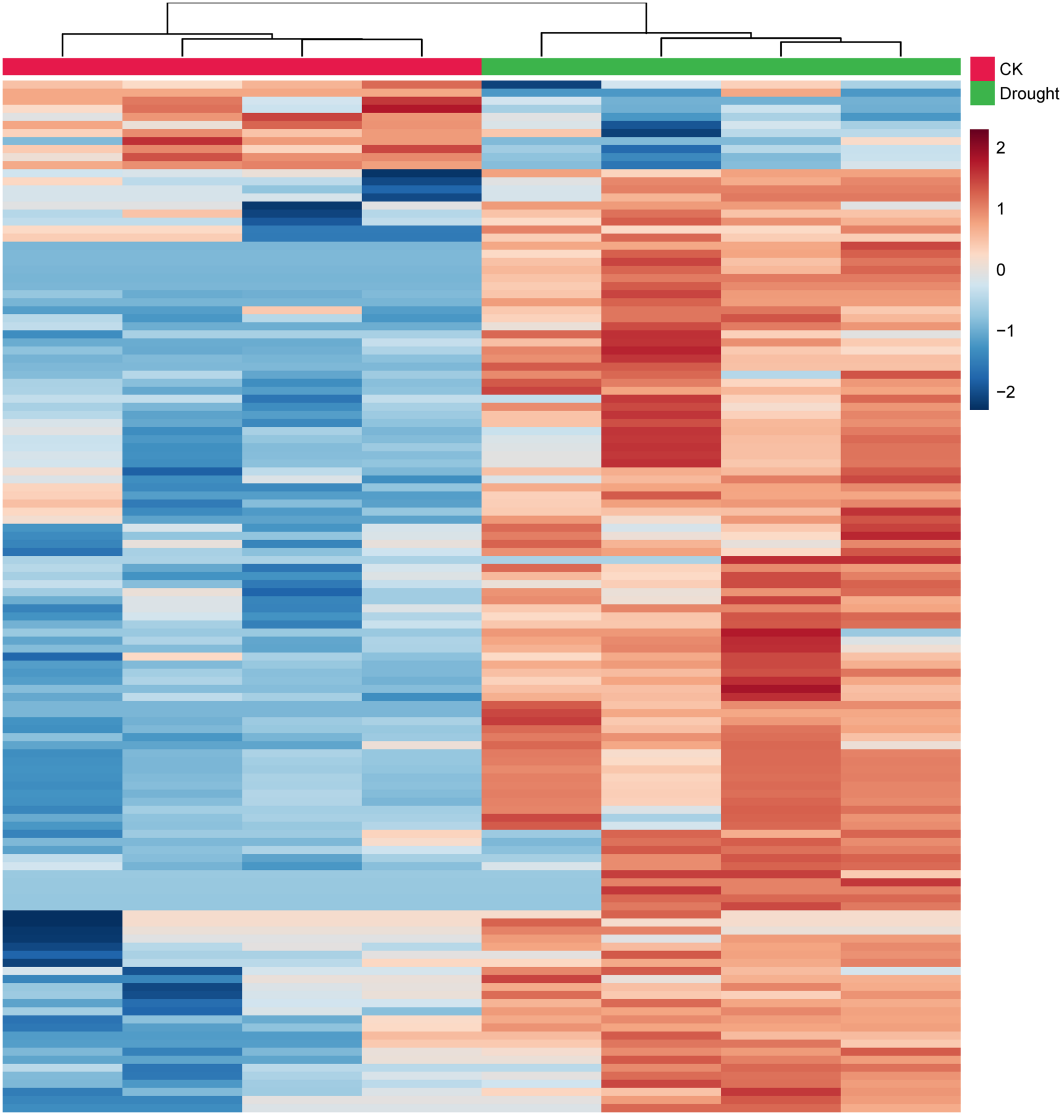
Heatmap clustering of the relative contents of the 129 differential metabolites of *C. breviculmis*. Each row represents a metabolite and each column represents a sample. The warmer color indicates higher content and the colder color denotes lower content.

Drought stress not only changed the biological endpoints (e.g., plant height, leaf fresh weight, MDA content and soluble protein content), but also altered the metabolite contents in the leaves of *C. breviculmis*. In total, 1,375 metabolites in leaves of *C. breviculmis* were confirmed. Compared with control, the metabolite with fold change > 2.0 or < 0.5 and *p* < 0.05, is discriminated as a differential metabolite. Therefore, 129 differential metabolites were detected (Supplementary Table 1). The 129 differential metabolites were clustered into phytohormones, sugars, amino acids, lipids, nucleotides, benzenoids, phenylpropanoids and polyketides, and other molecules based on their comprehensive functions/pathways. As shown in Figure 3, the metabolites of control and drought stress are separated into two groups using hierarchical clustering analysis. Among the differential metabolites, there are three endogenous phytohormones up-regulated induced by drought stress, including jasmonic acid (3.64-fold), indole-3-carboxaldehyde (2.78-fold) and 3-methylindole (2.71-fold). There are also three nucleotides increased under drought treatment, including adenosine (2.51-fold), adenine (2.25-fold) and dihydrouracil (3.53-fold). Likewise, ten sugars increased, such as maltose (2.91-fold), sucrose (2.40-fold), galactose (2.32-fold), and neotrehalose (3.66-fold), etc. Drought stress also increased the content of 26 amino acids (8 protein amino acids and 18 non-protein amino acids), such as pipecolic acid (35.47-fold), proline (17.74-fold), glutamylphenylalanine (11.11-fold) and hydroxylidocaine (10.82-fold). The contents of 6 organic acids significantly changed under drought stress, 5 of which increased, including humulinic acid A (14.42-fold), malic acid (2.58-fold), succinic acid (4.15-fold), 4-pyridoxic acid (2.85-fold) and quinaldic acid (6.15-fold) and only 2-oxoglutarate (0.21-fold) decreased. Twenty-seven lipids were up-regulated by drought stress, such as linolenic acid (2.02-fold) and tsugaric acid B (3.12-fold). However, five lipids were down-regulated, such as arachidic acid (0.23-fold), behenic acid (0.33-fold). The contents of 9 benzenoids were significantly enhanced by drought treatment, and only three benzenoids were suppressed. All the contents of phenylpropanoids and polyketides were enhanced by drought stress.

### Relationships Between Metabolite Changes and Biological Endpoints

**Figure 4:**
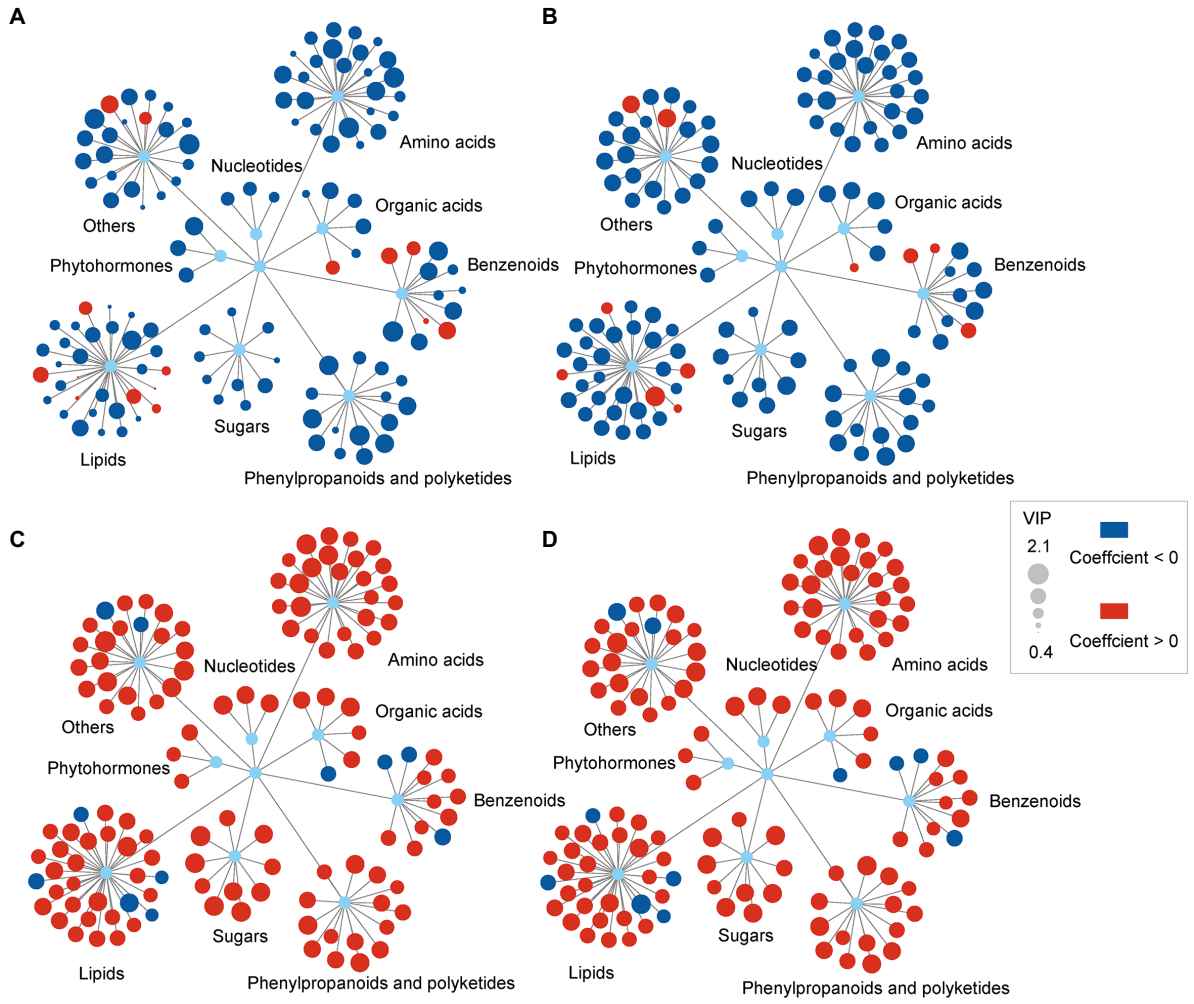
Relationships between the differential metabolite changes and biological endpoints of *C. breviculmis* induced by drought stress. **(A)** Plant height as the biological endpoint. **(B)** Leaf fresh weight as the biological endpoint. **(C)** Soluble protein content as the biological endpoint. **(D)** MDA as the biological endpoint. The red and blue circles represent active and passive coefficients, respectively. The sizes of the circles represent the variable importance projection (VIP) values.

The orthogonal partial least squares regression analysis was applied to clarify the linkages between biologic endpoints and changes in leaf metabolites. In the results of these analyses, the Variable Importance Projection (VIP) values were expressive of the effect of each metabolite on the biological endpoint, and the coefficient indicated the positive or negative relationship of the metabolite changes with the biological endpoint. Fifteen metabolites (e.g., arachidic acid, behenic acid, 2-oxoglutarate and thermophillin) showed positive relationships with plant height. In comparison, 114 differential metabolites, such as jasmonic acid, sucrose and malic acid, showed negative relationships with leaf fresh weight (Figure 4A). Eleven metabolites (e.g., monoethyl phthalate, arachidic acid, 2-oxoglutarate and thermophillin) demonstrated positive relationships with leaf fresh weight. In comparison, 118 differential metabolites, such as jasmonic acid, sucrose, malic acid, showed negative relationships with leaf fresh weight (Figure 4B). One hundred and eighteen metabolites, including phytohormone, sugars, amino acids, illustrated positive relationships with soluble protein content and MDA content. In contrast, 11 differential metabolites, belonging to lipids and benzenoids, showed negative relationships with soluble protein content and MDA content (Figures 4C,D). The relationships between metabolite changes and other biological endpoints were also analyzed, such as crown width, leaf length, leaf width and leaf dry weight. The results showed a similar trend to the relationships between metabolite changes and plant height (Supplementary Figure 1).

### Changes in Metabolic Pathways Under Drought

**Figure 5:**
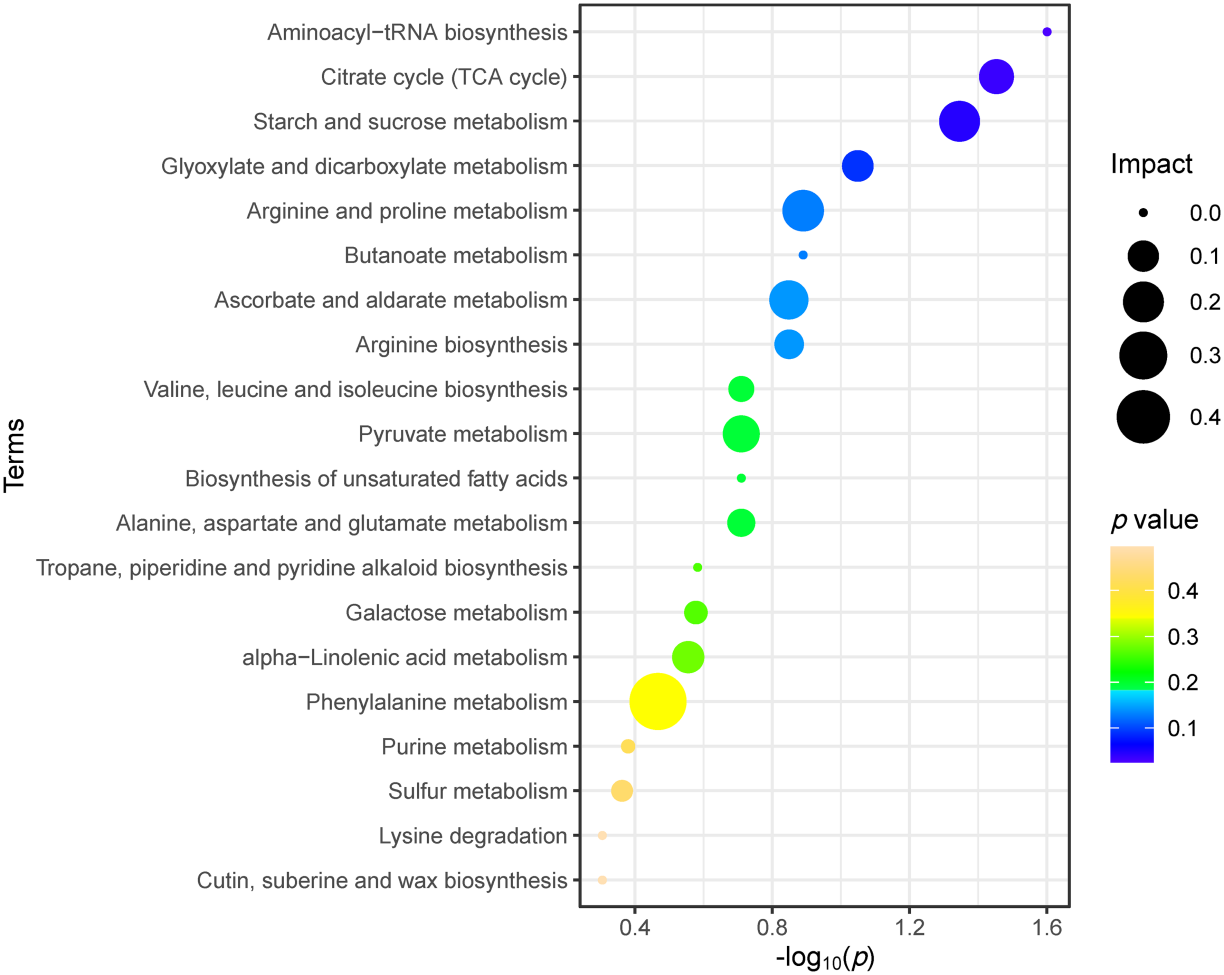
Top 20 metabolic pathways of *C. breviculmis* affected by drought stress *via* pathway analysis from differential metabolites. The pathway impact is computed from the pathway topological analysis and represented *via* the circle size. The *p* values obtained from pathway enrichment analysis showed both by the *x*-axis (−log10) and color.

**Figure 6:**
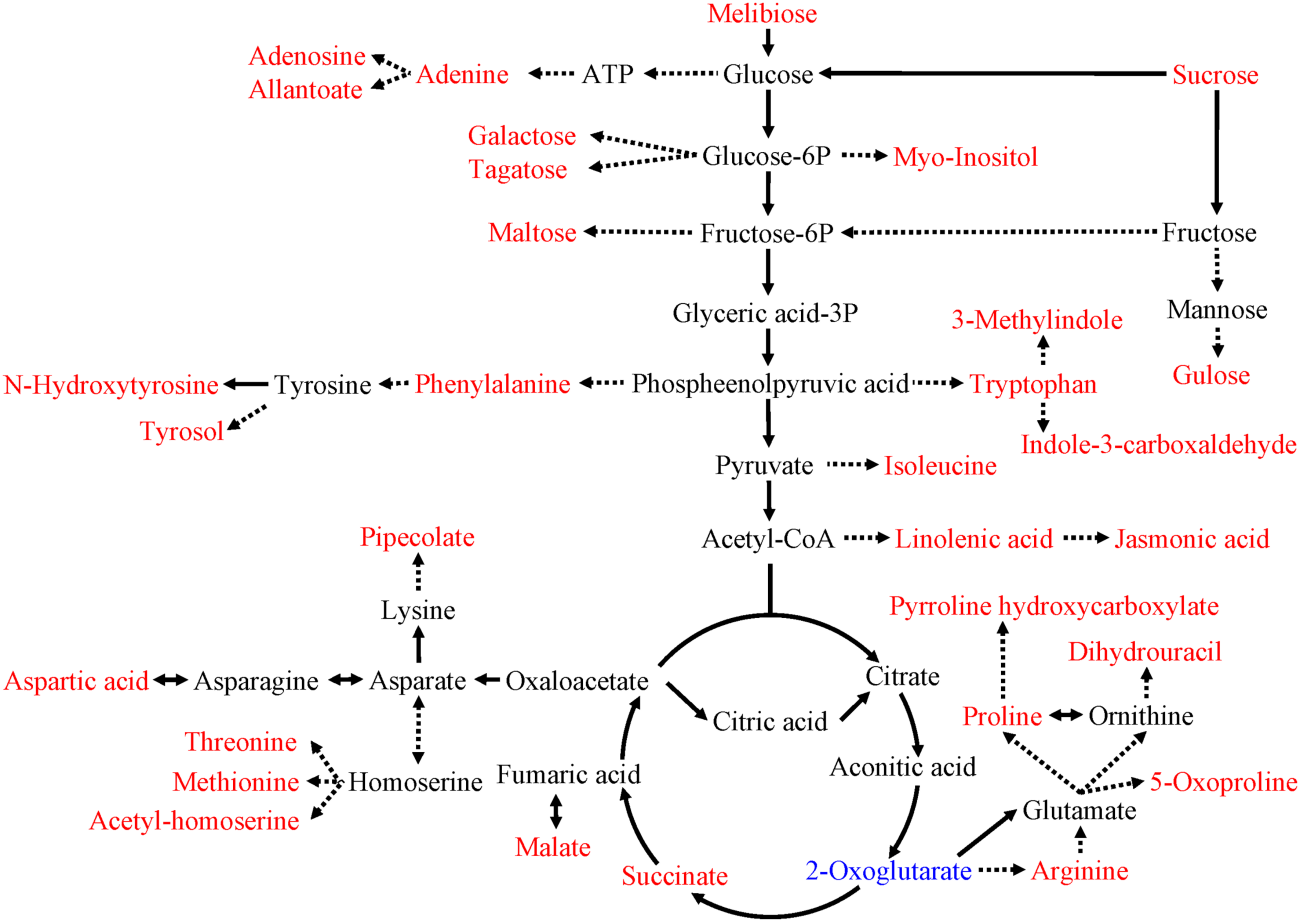
Effects of drought stress on the central metabolic pathways of *C. breviculmis*. The metabolites in the red or blue text were the differential metabolites detected in the present work. The words in the red/blue represent up-regulation/down-regulation of metabolites under drought treatments, respectively. Arrows with full/dotted lines denote the direct/indirect reactions, respectively.

As shown in Figure 5, the metabolic pathway analysis showed that drought stress affected the Aminoacyl-tRNA biosynthesis (0, 1.60), citrate cycle (TCA cycle; 0.13, 1.45), starch and sucrose metabolism (0.20, 1.35). The content of sugars and sugar alcohols increased significantly, such as melibiose, sucrose, galactose, tagatose, maltose, glucose and inositol. The content of amino acids and their derivatives also increased significantly, such as aspartic acid, arginine, threonine, methionine, phenylalanine (including its derivatives N-hydroxytyrosine and tyrosol), tryptophan (including its derivatives 3-methylindole and indole-3-carboxaldehyde; Figure 6). Similarly, the content of lipids and their derivatives significantly improved, such as linolenic acid and its jasmonic derivatives (Figure 6).

## Discussion

To achieve optimal growth and reproduction, plants require both water and nutrients with an appropriate amount to fulfill their essential needs. It has been reported that the drought-induced decrement in plant growth can be related to the variations in oxidative status, nutrition balance, hormone secretion, protein deterioration/synthesis, enzyme activity and the secondary metabolism (Jabeen et al., 2021).

### The Adverse Effects of Drought Stress on the Growth of *Carex breviculmis*

Drought inhibited the growth of *C. breviculmis*, leading to a decrease in plant height, crown breadth, leaf length and leaf width. The inhibition strength increased with the progress of the drying period. Some studies have also reported that plant height, leaf width and leaf length significantly decreased under drought conditions (Bhusal et al., 2020). As drought inhibited the growth of *C. breviculmis*, the leaf fresh weight and total fresh weight were also reduced. In *Matthiola incana*, the plant height, stem fresh weight, stem dry weight, root fresh weight and root dry weight significantly decreased (Jafari et al., 2019). Consistent with Du et al. (2020) study, the leaves were more sensitive to the soil moisture change and the leaf weight decreased more than the roots, leading to a higher root/shoot ratio under drought treatment.

### Drought Stress Enhanced Most of the Differential Primary Metabolites

Drought stress significantly increased most of the primary metabolites of *C. breviculmis*. Soluble sugar is a critical energy and carbon source in organisms and participates in many procedures of plant metabolism. Similar to previous studies, sugars and polyols (e.g., melibiose, sucrose, fructose, glucose, galactose, trehalose, tagatose, glucose, maltose, arabitol, ononitol, galactitol and myo-inositol) showed a significant increase in response to water deficit (Guo et al., 2018). The increases in these sugars may offer a primary defense against further water loss (Bowne et al., 2012). Sucrose is the main product of photosynthesis. Drought could increase sucrose phosphate synthase activity, resulting in an increase in sucrose content (Xu et al., 2015). The increase in sucrose may lead to a rise in its derivatives such as fructose and glucose which have been proved to assist in dehydration tolerance of plants by osmoprotection (Urano et al., 2009). In extremely dehydrated states, non-reducing sugars and oligosaccharides could be a water replacement, providing a hydration shell around proteins (Hoekstra et al., 2001). The accumulation of polyols such as myo-inositol may facilitate osmotic adjustment and support redox control (Llanes et al., 2018). In contrast, the sugars and polyols accumulated under drought stress, the organic acids, including the TCA cycle intermediates, such as succinic and malic acid, also increased (Sanchez et al., 2012). However, the 2-oxoglutarate decreased in our study, which was also found in leaves of some plants under drought stress (Sicher and Barnaby, 2012). Otherwise, arginine, a derivative of 2-oxoglutarate, increased under drought stress, which could provide defense against short-time drought stress in wheat seedlings (Hasanuzzaman et al., 2018). Metabolic pathway analysis showed that drought stress significantly affected the TCA cycling, starch and sucrose metabolism. In plant growth, carbohydrates play a vital role as the primary energy metabolism substances and have a significant regulatory effect on plant growth and development, metabolic regulation and anti-stress. A possible explanation for the increase in sugars is that plant growth drops before photosynthesis under drought stress, resulting in an excess of carbon skeletons which can be directed to osmolytes production (Gurrieri et al., 2020).

In addition to the accumulation of sugars and polyols, up- and down-regulation of amino acid metabolism arise in response to environmental stresses (Han et al., 2019, 2021). The three aromatic amino acids (tryptophan, phenylalanine and tyrosine) are synthesized *via* the shikimate pathway and serve as precursors for wide-ranging secondary metabolites, such as indole acetate, glycosides and terpenoids (Less and Galili, 2008). Tryptophan and phenylalanine increased significantly under drought stress, which has been reported in many plants (Khan et al., 2019). The high level of tryptophan may lead to the accumulation of indole-3-carboxaldehyde and 3-methylindole, which act as plant growth inhibitors. The higher phenylalanine content may also lead to more tyrosol, which is considered a powerful antioxidant agent (Mechri et al., 2020). Our results showed that the proline level remarkably enhanced under drought stress, similar to the drought tolerance study in soybean (Moloi and van der Merwe, 2021). Proline, a derivative of glutamate, is participated in osmotic adjustments and cell organelle safety. Exogenous proline can help remove free radicles, therefore keeping plants from harmful effects of reactive oxygen species induced by drought stress (Seki et al., 2007). Membrane deterioration was lessened due to lower MDA production under the application of proline because proline promotes the activity of the antioxidant defense system (Wani et al., 2012). 5-Oxoproline, another derivative of glutamate, also increased under drought stress, which could improve the drought tolerance ability and increase yield under water-deficit stress (Jiménez-Arias et al., 2019).

Apart from the changes in aromatic amino acids, the branched-chain amino acids (BCAAs) varied under water-deficit conditions. Isoleucine content was significantly increased under drought stress, which was similarly reported in many plants under drought stress and in response to other osmotic stress conditions (Khan et al., 2019). The increase in isoleucine may be due to a possible stress-related function in the constitution of the jasmonate-isoleucine conjugate, which improved in response to osmotic stress (Urano et al., 2017). Our results showed that aspartic acid increased under drought stress, which could significantly decrease the permeability of plasma membrane and MDA content (Wang and Yang, 2015).

The allantoic acid was enhanced by drought stress, which also found to accumulate in roots, shoots and leaves of *Phaseolus vulgaris* under drought stress (Alamillo et al., 2010). The increase in allantoic acid corresponds with the rise of adenine, leading to a rise of adenosine content. The pipecolate, a lysine-derived non-protein heterocyclic amino acid, increased under drought stress. Pipecolate significantly increased under osmotic stresses, metal stresses and nutrient deficiencies, indicating pipecolic acid plays a vital role in plant systemic acquired counteraction against biotic elicitors (Zhao et al., 2021). Threonine, methionine and acetyl-homoserine, three derivatives of homoserine, also increased under drought stress. Our results are consistent with the previous studies that the content of threonine and methionine significantly increased under drought stress, and the accumulation of threonine and methionine enhanced plant stress resistance (You et al., 2019). Threonine could serve as synthetic substrates of osmotic regulatory substances; therefore, the accumulation of threonine plays a significant role against abiotic stress (Chang et al., 2020). Methionine act as an effective regulator of plant growth and development under environmental cues, including drought stress (You et al., 2019), and acetyl-homoserine is likely involved in the biosynthesis of proline (Blankenship, 2004).

The soluble protein content significantly increased under drought stress. When encountering drought or other stresses, the synthesis rate of protein in plants may reduce. However, the synthesis of some original proteins (namely stress-induced proteins, such as dehydrin-like proteins) may be induced or up-regulated to maintain the osmotic potential of cells to keep a certain turgor and thereby guarantee the usual physiological procedures such as cell growth, stomatal opening, photosynthesis and so on (Li et al., 2010). Similar to previous studies, the soluble protein content arose along with the stress aggravating (Hou et al., 2021). Metabolic pathway analysis showed that drought stress significantly affected the aminoacyl-tRNA biosynthesis, which is closely related to the significant changes in protein metabolism and the massive accumulation of amino acids, therefore the phenotypic damage in *C. breviculmis*. Compared with well-watered plants, the activity of intracellular proteases and endopeptidases increased significantly under drought conditions, resulting in increased protein degradation and amino acid reactivation, which in turn increased amino acid content. Due to the need for osmotic regulation and the synthesis of dehydrin-like proteins, the supply of amino acids required for plant growth is insufficient, manifesting as the amino acid content negatively correlated with plant growth indicators and positively correlated with soluble protein content and MDA content.

In plants, fatty acids and derived metabolites are also identified as signaling molecules central to diverse biological processes. Exogenous and endogenous unsaturated fatty acids and fatty acid metabolites can significantly change plant gene expression and metabolism to affect the consequence of plant-biotic and plant-abiotic interactions (Upchurch, 2008). Linolenic acid, a kind of polyunsaturated fatty acid, is a major precursor for messengers containing jasmonic acid. We found that the content of linolenic acid increased by the drought stress, which is in agreement with the previous studies on tree peony (Zhao et al., 2019) and tobacco (Yang et al., 2017). The increase in linolenic acid may be due to the over-expression of genes related to linolenic acid metabolism (Huang et al., 2019). However, studies reported a drought-induced decrease in linolenic acid, reflecting damage instead of defense (Zhang et al., 2005). Our results showed that drought stress increased the content of arachidonic acid, which agrees with the study of Saheri et al. (2020). Arachidonic acid can perform as a signal molecule that not only sparks fatty acid-mediated defenses, but also adjusts the general stress transcriptional network in addition to the jasmonate-biosynthetic pathway (Savchenko et al., 2010). Our result corresponds with Toumi et al. (2008), that drought can increase total lipid content, which is manifested as lipid changes are inversely associated with growth indices and positively correlated with cell damage.

### Drought Stress Enhanced Most of the Differential Secondary Metabolites

In addition to the changes of primary metabolites for essential functions, a large number of secondary metabolites increased/decreased under drought stress. MDA is a highly reactive tri-carbon dialdehyde generated as a byproduct of polyunsaturated fatty acid peroxidation and arachidonic acid metabolism, recognized as a biomarker of oxidative stress. Our results are consistent with the study of Jaafar et al. (2012), that drought stress promoted the content of MDA closely related to linolenic acid metabolism and tryptophan metabolism.

Phytohormones play important roles as plant growth regulators, influencing a wide range of morphological, physiological and biosynthetic processes, especially stress tolerance. Jasmonic acid could adjust the stomatal closure and invoke defense mechanisms of plants in response to various environmental stresses such as salinity, low temperature, elemental toxicity and especially drought-induced oxidative damages (Mahmood et al., 2012). The jasmonic acid content also increased under drought stress in other plants (Wang et al., 2021). Likewise, the indole-3-carboxaldehyde and 3-methylindole content enhanced under drought stress in our experiment. The indole-3-carboxaldehyde plays an essential role as a growth inhibitor of seed germination, lateral bud growth, root and shoot growth (Suzuki et al., 2019). Similarly, 3-methylindole, functioning as an indole, could suppress plant growth (Patiño et al., 2018).

Benzaldehyde, a key module candidate for growth inhibition, increased with the aggravation of drought stress (Timmusk et al., 2014), which is also observed in our study. 10-Paradol, an antioxidant with antimicrobial activity, was also found to increase under water deficit (Ackah et al., 2021). Under drought stress, avenalumic acid also accumulated, which is readily hydrolyzed on contact with water to produce the corresponding strong antioxidant avenanthramides (Peterson, 2001). The batatasin I was also increased under drought stress, considered an inhibitor of plant growth (Hu et al., 2015). The antioxidative coumarins, 3-hydroxycoumarin and 5-methoxyseselin, increased under drought stress, and the increase in 3-hydroxycoumarin may be due to the accumulation of sucrose (Kim et al., 2020). We also found drought stress enhanced the content of cyclocurcumin, which has been found recently to be an antioxidant and could scavenge free radicals (Li et al., 2018). Isomucronulatol exhibited noticeable ABTS^+^ scavenging activity, which also increased under drought stress in our study (Öztürk et al., 2011). In agreement with former studies, resveratrol, a robust defense antioxidant, accumulated in leaves and fruits that encountered biotic stresses (fungal infection) or abiotic stresses (drought, UV irradiation, wounding and chemical treatments; Corso et al., 2015).

One mechanism plant adopted for surmounting the water-deficit effects might be the accumulation of reconcilable osmolytes, such as proline, soluble sugars. Our results showed that almost all the sugars, amino acids, organic acids, phytohormones, nucleotides, phenylpropanoids and polyketides of differential metabolites are negatively correlated with plant height and leaf fresh weight, but positively correlated with soluble protein content and MDA content. The organic osmotic regulating substances are usually of low molecular mass, highly soluble and have little cell toxicity. They can retain the normal osmotic pressure, enzyme activities and membrane architecture. Moreover, most osmotic regulators fail to protect proteins under severe drought stress. However, only soluble sugars can substitute water molecules and constitute hydrogen bonds with proteins to keep the specific structure and activity of proteins (Toumi et al., 2008).

## Conclusion

In the differential metabolites, drought increased the content of most primary and secondary metabolites, and almost all the sugars, amino acids, organic acids, phytohormones, nucleotides, phenylpropanoids and polyketides were negatively correlated with plant height and leaf fresh weight, while positively correlated with soluble protein content and MDA content. Metabolic pathway analysis showed that drought stress significantly affected aminoacyl-tRNA biosynthesis, TCA cycling, starch and sucrose metabolism. Under drought stress, *C. breviculmis* mainly tolerates drought stress by adjusting the above metabolic pathways.

## Data Availability Statement

The original contributions presented in the study are included in the article/supplementary material, further inquiries can be directed to the corresponding author/s.

.

## Author Contributions

ZM conducted data analysis and wrote the original manuscript. ZM, YM, LD, XL, HZ, and LZ conducted the experiment. PL, HC, WJ, XZ, and YW assisted the experiment preparation. TH, HL, and ZM conceived the project. ZM and CZ supervised the studies and revised the manuscript. All authors contributed to the article and approved the submitted version.

## Funding

This work was supported by the National Natural Science Foundation of China (No. 31700368 and 31500193), Scientific and Technological Project of Henan Province (No. 212102110437), High-level Talent Scientific Research Start-Up Project of Henan Institute of Science and Technology (No. 2018017), and The Key Program of Higher Education of Henan Province (21A180010).

## Conflict of Interest

The authors declare that the research was conducted in the absence of any commercial or financial relationships that could be construed as a potential conflict of interest.

## Publisher’s Note

All claims expressed in this article are solely those of the authors and do not necessarily represent those of their affiliated organizations, or those of the publisher, the editors and the reviewers. Any product that may be evaluated in this article, or claim that may be made by its manufacturer, is not guaranteed or endorsed by the publisher.

## Supplementary Material

Supplementary Table 1 The details of 129 differential metabolites in Carex breviculmis under drought stress.
