# Supplementary material for: Combining Metabolic Analysis With Biological Endpoints Provides a View Into the Drought Resistance Mechanism of Carex breviculmis
Source: Front Plant Sci. 2022 Jul 7;13:945441. doi: 10.3389/fpls.2022.945441 (PMC9380063; doi:10.3389/fpls.2022.945441)
Supplement: Supplementary file 2 [file Table_2.pdf]

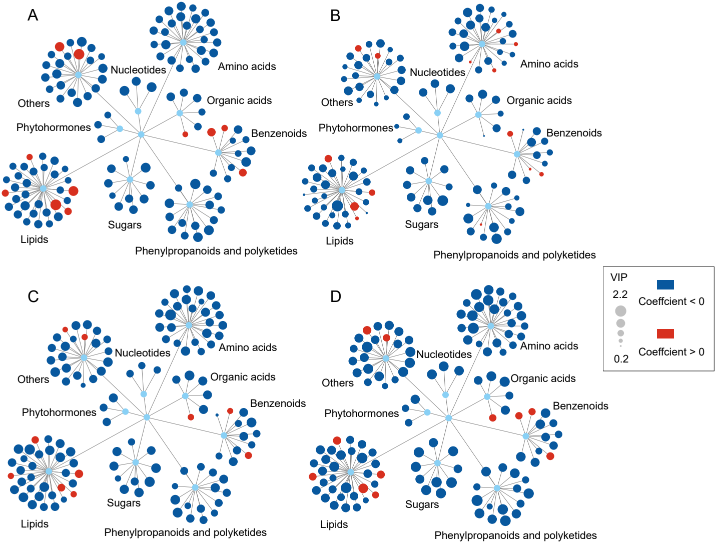

Supplementary Figure 1. Relationships between differential metabolite changes and biological endpoints of *C. breviculmis* under drought stress. (A) Crown width as the biological endpoint. (B) Leaf dry weight as the biological endpoint; (C) Leaf length as the biological endpoint. (D) Leaf width as the biological endpoint. The red and blue circles represent active and passive coefficients, respectively. The sizes of the circles represent the VIP values.
